# Biocompatible 5-Aminolevulinic Acid/Au Nanoparticle-Loaded Ethosomal Vesicles for In Vitro Transdermal Synergistic Photodynamic/Photothermal Therapy of Hypertrophic Scars

**DOI:** 10.1186/s11671-017-2389-x

**Published:** 2017-12-15

**Authors:** Zheng Zhang, Yunsheng Chen, Jiayue Ding, Chunlei Zhang, Amin Zhang, Dannong He, Yixin Zhang

**Affiliations:** 1 Department of Plastic and Reconstructive Surgery, Shanghai Ninth People’s Hospital, School of Medicine, Shanghai Jiao Tong University, 639 Zhizaoju Rd, Shanghai, 200011 People’s Republic of China; 2 Institute of Nano Biomedicine and Engineering, Shanghai Engineering Research Center for Intelligent Instrument for Diagnosis and Therapy, School of Biomedicine Engineering, Shanghai Jiao Tong University, 800 Dongchuan Rd, Shanghai, 200240 People’s Republic of China; 3 Department of Plastic Surgery, Lishui People Hospital, 15 dazhong Rd, Lishui, Zhejiang 323000 China; 4 Shanghai National Engineering Research Center for Nanotechnology, 245 East Jiangchuan Road, Shanghai, 200237 People’s Republic of China

**Keywords:** Hypertrophic scars, Ethosomal vesicles, 5-Aminolevulinic acid, Au nanoparticles, Synergistic photodynamic/photothermal therapy, Transdermal

## Abstract

Biocompatible 5-aminolevulinic acid/Au nanoparticle-loaded ethosomal vesicle (A/A-ES) is prepared via ultrasonication for synergistic transdermal photodynamic/photothermal therapy (PDT/PTT) of hypertrophic scar (HS). Utilizing ultrasonication, Au nanoparticles (AuNPs) are synthesized and simultaneously loaded in ethosomal vesicles (ES) without any toxic agents, and 5-aminolevulinic acid (ALA) is also loaded in ES with 20% of the entrapment efficiency (EE). The prepared A/A-ES displays strong absorbance in 600-650 nm due to the plasmonic coupling effect between neighboring AuNPs in the same A/A-ES, which can simultaneously stimulate A/A-ES to produce heat and enhance quantum yields of reactive oxygen species (ROS) by using 632 nm laser. In vitro transdermal penetrability study demonstrates that A/A-ES acts as a highly efficient drug carrier to enhance both ALA and AuNPs penetration into HS tissue*.* Taking human hypertrophic scar fibroblasts (HSF) as therapeutic targets, synergistic PDT/PTT of HS indicates that A/A-ES could enhance quantum yields of ROS by photothermal effect and localized surface plasmon resonance (LSPR) of AuNPs, resulting in a high level of apoptosis or necrosis. In a word, the prepared A/A-ES shows a better synergistic PDT/PTT efficiency for HSF than the individual PDT and PTT, encouraging perspective for treatment of HS.

**Electronic supplementary material:**

The online version of this article (10.1186/s11671-017-2389-x) contains supplementary material, which is available to authorized users.

## Background

Hypertrophic scar (HS), a common and inevitable problem after cutaneous dermal injury, has a much thicker fibrotic dermis than normal skin [1, 2]. Histopathologically, HS displays the increases of hypertrophic scar fibroblasts (HSF), which are arranged in wavy patterns, oriented to the epithelial surface and form nodular structures [3]. Although various treatments are available clinically, there are many challenges in HS treatments due to the numerous limitations. Intralesional injection therapy is widely used for clinical practices. However, it is limited by both uncomfortable operations and side effects, such as permanent hypopigmentation and skin atrophy [4]. Pressure therapy is limited for side effects, such as tissue ischemia as well as decreasing tissue metabolism [5]. To overcome these limitations, laser therapy serves as a topical and non-invasive modality that has been developed and applied in HS treatments for more than 25 years, by taking advantages of the laser irradiation [6]. Generally, laser therapy can be divided into photodynamic therapy (PDT) and photothermal therapy (PTT) based on the different principles.

PDT has been used to treat HS with the advantages of its high selectivity and few side effects [7]. Its principle evolves two steps: (a) photosensitizers preferentially aggregate in HSF and (b) under the irradiation of an appropriate laser, photosensitizers produce cytotoxic reactive oxygen species (ROS) which lead to the apoptosis of HSF [8, 9]. Among various photosensitizers, 5-aminolevulinic acid (ALA) is proven to be an excellent candidate for local treatment modality in dermatology without significant side effect. Therefore, ALA-based PDT (ALA-PDT) has been widely used in HS treatment with marketing permission from the US Food and Drug Administration in 2010 [10]. However, its efficiency is controversial for two limitations: (a) the poor penetrability of ALA into both HS tissue and HSF and (b) the low quantum yields of ROS. In order to produce a marked effect, a high-dose ALA or high-level laser is applied in clinic. Unfortunately, high-dose ALA leads to damage of the sebaceous gland and epidermis, and high-level laser tends to result in healthy tissues injured. Therefore, much attention has been paid to enhance penetrability of ALA and quantum yields of ROS in PDT treatment of HS. Recently, ethosomal vesicles (ES), a specifically designed liposome, are found to be able to overcome the barrier in HS for topical delivery and achieve significant progress [11, 12]. In our prior work, the prepared ALA-loaded ES (ALA-ES) is capable of delivering much more ALA into HS compared with traditional hydroalcoholic solution system [13]. Therefore, ES can enhance penetrability of ALA to improve PDT efficacy of HS. Meanwhile, a new synergistic treatment modality, which combines PDT with PTT, holds the promise to enhance both the quantum yields of ROS and the treatment efficacy of HS.

PTT is also an extraordinary theranostic approach for various diseases [14, 15]. Up to now, it has been successfully applied in clinical treatment of HS [16]. Its mechanism evolves harvesting light energy, generating heat, and then resulting in tissue vaporization, coagulation, HSF apoptosis, and collagen denaturation. However, PTT has severe side effects in HS treatment, such as oozing, ulceration, and burning discomfort, due to its poor selectivity toward HS tissue with high-level laser [4]. Recently, PTT, bridging nanotechnology, has been regarded as a potential HS treatment with highly selective and minimally invasive for the photothermal effect. And more importantly, based on Au nanoparticles (AuNPs) as effective photo-adsorbing agents, PTT has been confirmed to enhance quantum yields of ROS for two reasons: (a) thermal PDT significantly increases apoptotic cell death through enhancing generation of ROS in a temperature-dependent manner, and [17] (b) AuNPs can conjugate with ALA and enhance quantum yields of ROS due to localized surface plasmon resonance (LSPR) [18, 19]. Therefore, ALA/AuNP-based synergistic photodynamic/photothermal therapy (PDT/PTT) holds the promise to overcome current limitations of both PDT and PTT in HS treatment.

Recent, AuNP-based synergistic PDT/PTT has been widely used in various cancer therapies by injection ways [20, 21]. Different from cancers, HS is suitable for using topical administration [22]. However, the collagen bundles in HS dermis present great barriers to the penetration of ALA and AuNPs, which restricts the PDT/PTT synergistic treatment efficiency for HS. Therefore, how to make ALA and AuNPs simultaneously penetrate into HS is critical to synergistic PDT/PTT with maximum therapeutic efficacy and minimum side effect [23, 24]. Furthermore, a suitable ALA/AuNP-based synergistic PDT/PTT should also satisfy the following conditions: (a) AuNPs can generate heat by He-Ne laser which is used in ALA-PDT, and (b) the delivery system should be high biocompatible. However, the reported various photosensitizers/AuNPs cannot be applied by a topical transdermal delivery and HS treatment for penetrability and poor biocompatibility [25].

Herein, ALA/AuNP-loaded ES (A/A-ES) with excellent biocompatibility and penetrability is developed for synergistic PDT/PTT of HS in this work. The biocompatible A/A-ES is prepared by both AuNPs and ALA-loaded ES via an ultrasonication process without any toxic agent. The prepared A/A-ES shows a strong absorbance in the range of 600–650 nm, as a result of the plasmonic coupling between neighboring AuNPs co-loaded in A/A-ES. This enables the use of He-Ne laser to stimulate A/A-ES to simultaneously generate heat and ROS, which could promote HSF apoptosis. A/A-ES displays excellent penetrability to simultaneously deliver ALA and AuNPs into HS in the in vitro study. At last, taking HSF as the target, in vitro efficiency PDT/PTT for HS is investigated by accumulation of intracellular protoporphyrin IX (PpIX), quantum yields of ROS, and apoptosis of HSF. Furthermore, the penetrability into HSF is also observed by TEM. Due to the synergistic effect, A/A-ES facilitates both ALA and AuNPs to simultaneously penetrate into HS and HSF, causing a higher level of cell apoptosis compared to individual PTT or PDT. In a word, A/A-ES is a promising transdermal delivery system for topical ALA and AuNP administration, has great potential in synergistic PDT/PTT of HS, and opens a new window for HS treatment.

## Results and Discussions

### The Characterization of A/A-ES

Ultrasonication was the key parameter in preparing A/A-ES for two reasons: (a) AuNPs could be formed via ultrasonication without any toxic agent, which endowed A/A-ES with biocompatibility; (b) ultrasonication could rearrange the lipid bilayers to form more vesicles with small sizes and relatively lager internal cores, which could load more ALA and AuNPs. In this work, AuNPs were formed as described in the following schemes: (a) highly reactive H• and OH• radicals were generated within the bubbles by the homolysis of H_2_O (Eq. 1), (b) the oxidizing radicals H• could abstract the alpha H of CH_3_CH_2_OH and form a reducing radical CH_2_•CH_2_OH (Eq. 2), and (c) during a pyrolysis within the bubbles, the radical CH_2_•CH_2_OH could reduce Au^3+^ to form AuNPs (Eq. 3) [26].

1 $$ {\mathrm{H}}_2\mathrm{O}\to \mathrm{H}\bullet +\mathrm{OH}\bullet $$

2 $$ \mathrm{H}\bullet +{\mathrm{CH}}_3{\mathrm{CH}}_2\mathrm{OH}\to {\mathrm{CH}}_2\bullet {\mathrm{CH}}_2\mathrm{OH}+{\mathrm{H}}_2 $$

3 $$ {\mathrm{Au}}^{3+}+{\mathrm{CH}}_2\bullet {\mathrm{CH}}_2\mathrm{O}\mathrm{H}+\mathrm{OH}\bullet \to \mathrm{AuNPs}+{\mathrm{CH}}_3{\mathrm{CH}}_2\mathrm{O}\mathrm{H}+{\mathrm{H}}_2\mathrm{O} $$

**Fig. 1:**
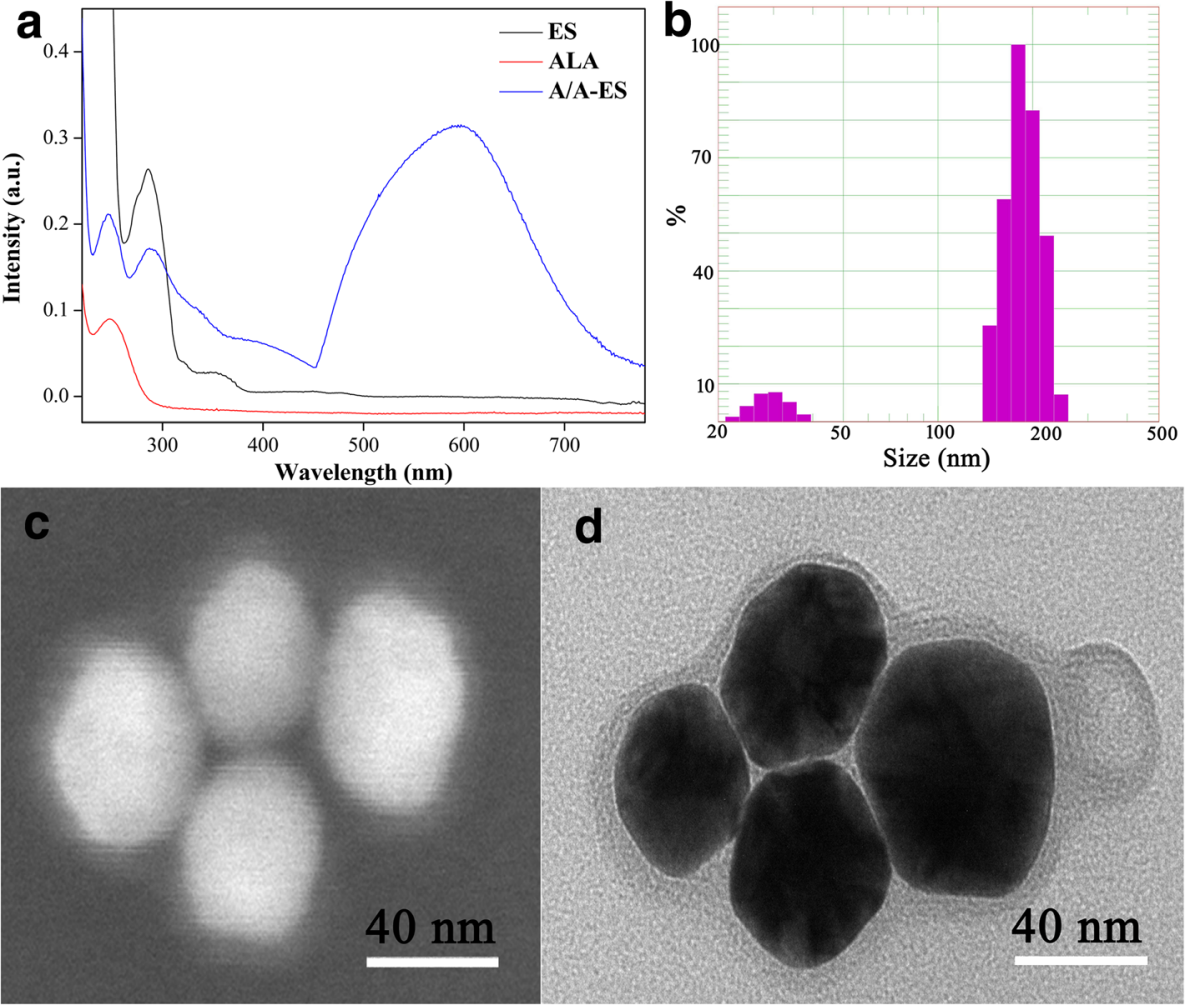
**a** UV-Vis spectra of ES, ALA, and A/A-ES. **b** The size distribution of prepared A/A-ES. **c**, **d** The SEM and TEM images of A/A-ES

At first, A/A-ES was verified by UV-Vis (Fig. 1a). It had a strong absorbance in the range of 600–650 nm, as a result of the plasmonic coupling between neighboring AuNPs in the same A/A-ES [20]. Therefore, it could use 632-nm laser irradiation to simultaneously PDT and PTT for HS. Furthermore, A/A-ES exhibited a relatively narrow size distribution and the average size was 166 ± 83 nm, according to DLS analysis in Fig. 1b. Interestingly, two size distributions were attributed to unloaded AuNPs and A/A-ES. Also, the great difference between two distributions suggested that the amount of A/A-ES was much more than that of unloaded AuNPs. The PDT efficiency was depended on the amount of ALA loaded in A/A-ES. Benefiting from the transmembrane pH gradient active loading method, EE of ALA was of 20%, which was higher than the ones in reported works (less than 10%) [27]. The morphology of A/A-ES was also studied. On SEM images (Fig. 1c), A/A-ES appeared as intact spherical lamellar vesicles with size at 200 nm, and AuNPs could be clearly observed and loaded in ES. Besides the AuNPs, the lamellas extended to AuNP surface in Fig. 1d, which was the characteristic of ES [28, 29]. Furthermore, the prepared A/A-ES loading different numbers of AuNPs had the similar sizes in Additional file 1: Figure S1. Therefore, A/A-ES was adjusted into the stable and deformable structure under ultrasonication, which facilitates A/A-ES to squeeze through narrow space in HS. To sum up, A/A-ES was successfully prepared with 20% EE of ALA and strong absorbance at 600–650 nm. Its morphology would also be very conducive to penetrability, which was in consistence with the in vitro PDT/PTT study in followings.

### In Vitro Transdermal Penetrability Study of A/A-ES

**Fig. 2:**
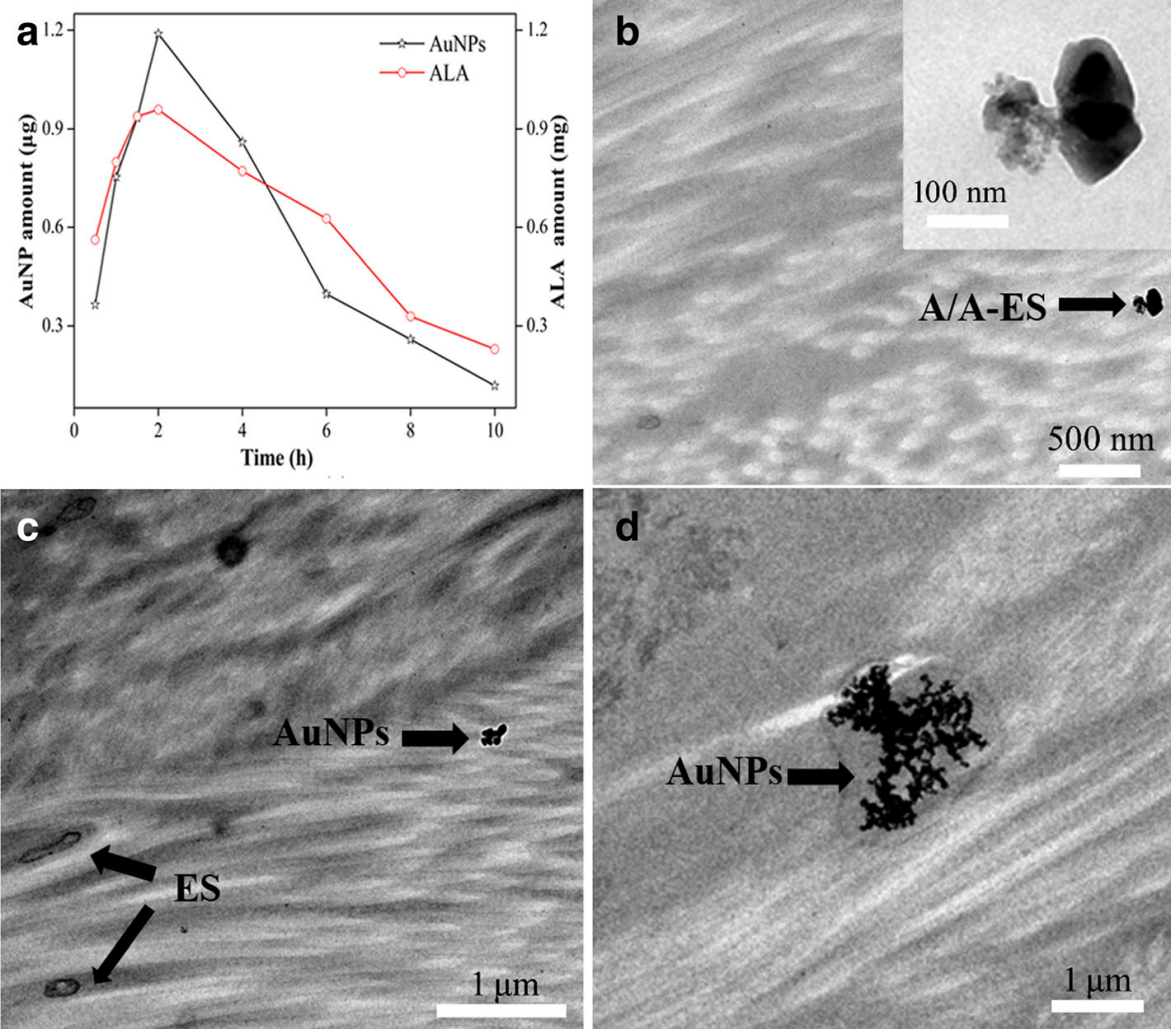
**a** the retention amount of ALA and AuNPs. **b** The distribution of A/A-ES in HS tissue. **c** The distribution of ES and AuNPs in HS tissue. **d** AuNPs accumulating in HS tissue

The retention of A/A-ES was important parameters for evaluating the penetrability and treatment efficiency of A/A-ES. Therefore, the retention amount of both ALA and AuNPs in HS with different time was investigated by using Franz diffusion cells. As shown in Fig. 2a, both ALA and AuNPs rapidly reached the maximum retention in the first 2 h, due to the penetration enhancement function of ES. After reaching the maximum, the retentions both of ALA and AuNPs continuously declined because A/A-ES penetrated through the whole HS. The results indicated the A/A-ES had enough penetrability. Compared with applied dose of ALA (2 mg), 48% ALA was in HS tissue, which was in favor of PDT of HS. Furthermore, the same retention changes between ALA and AuNPs suggested that ALA and AuNPs were both loaded in ES as consistent with results of microscopes. According to the result, 2 h was a proper administration time for topical usage with the maximum retention amount of A/A-ES. In our previous works, ES had been regarded as a highly efficient drug carrier to enhance drug penetration into HS tissue [13]. Therefore, the distribution and action of A/A-ES in HS was also studied by using TEM in this work. As shown in Fig. 2b, A/A-ES, as intact structure, was found in dermis, indicating A/A-ES could stably penetrate through epidermis and into HS dermis. In the lower dermis shown in Fig. 2c, the ES and AuNPs were observed as a separation state, suggesting A/A-ES would release both ALA and AuNPs. Interestingly, AuNPs could be aggregative in dermis even though they were not loaded in ES. Furthermore, more AuNPs were found to accumulate in dermis in Fig. 2d, which could provide the plasmonic coupling between neighboring AuNPs to harvest light energy and generate heat. In brief, in vitro transdermal penetrability study demonstrated A/A-ES was a highly efficient drug carrier to enhance both ALA and AuNP penetration into HS tissue, and the aggregative AuNPs in dermis was in favor of generate heat [20]. Therefore, the A/A-ES displayed a great potential in synergistic PDT/PTT for HS.

### In Vitro PDT/PTT of HSF

#### Biocompatibility Assay

**Fig. 3:**
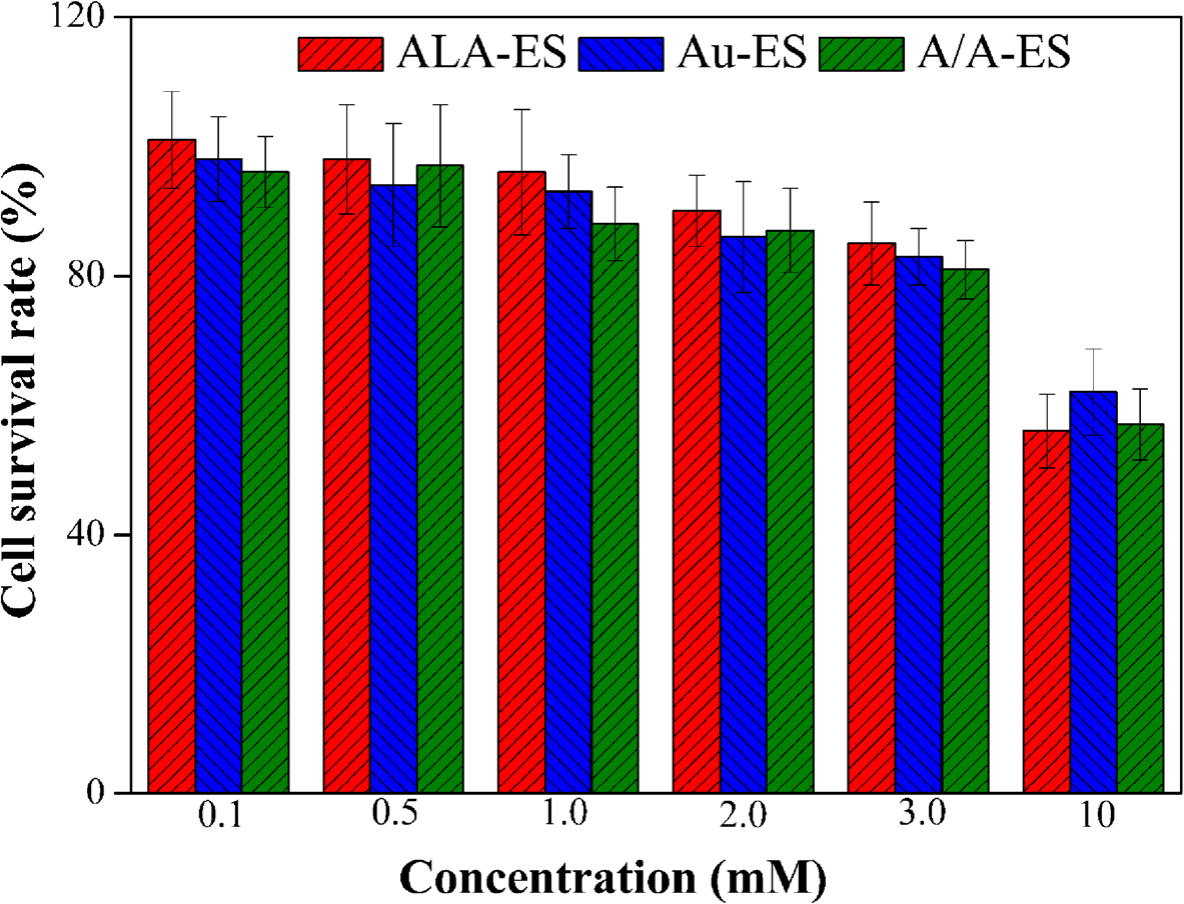
The cell viability of HSF upon treatment with ALA-ES, Au-ES and A/A-ES in the dark for 12 h

Although the biocompatibility of AuNPs had been well proven in reported work, the biocompatibility of A/A-ES to HSF should be also studied in this work [30, 31]. Different concentrations of ALA-ES, Au-ES, and A/A-ES (based on ALA concentrations from 0.1 to 10 mM, Au-ES was the same AuNP concentration as A/A-ES) were incubated with HSF for 12 h without irradiation. The result showed that there was no dark cytotoxicity in the concentrations of no more than 2.0 mM with cell survival rates more than 90%. When the concentrations were higher than 2.0 mM, a slight decrease in cell survival rates was detected. The results showed that A/A-ES had the excellent biocompatibility, and the PDT/PTT should be carried out at a concentration of 2.0 Mm in following studies (ca. 14% A/A-ES in culture mediums, *v*/*v*.) Fig. 3.

### PDT/PTT for HSF

**Fig. 4:**
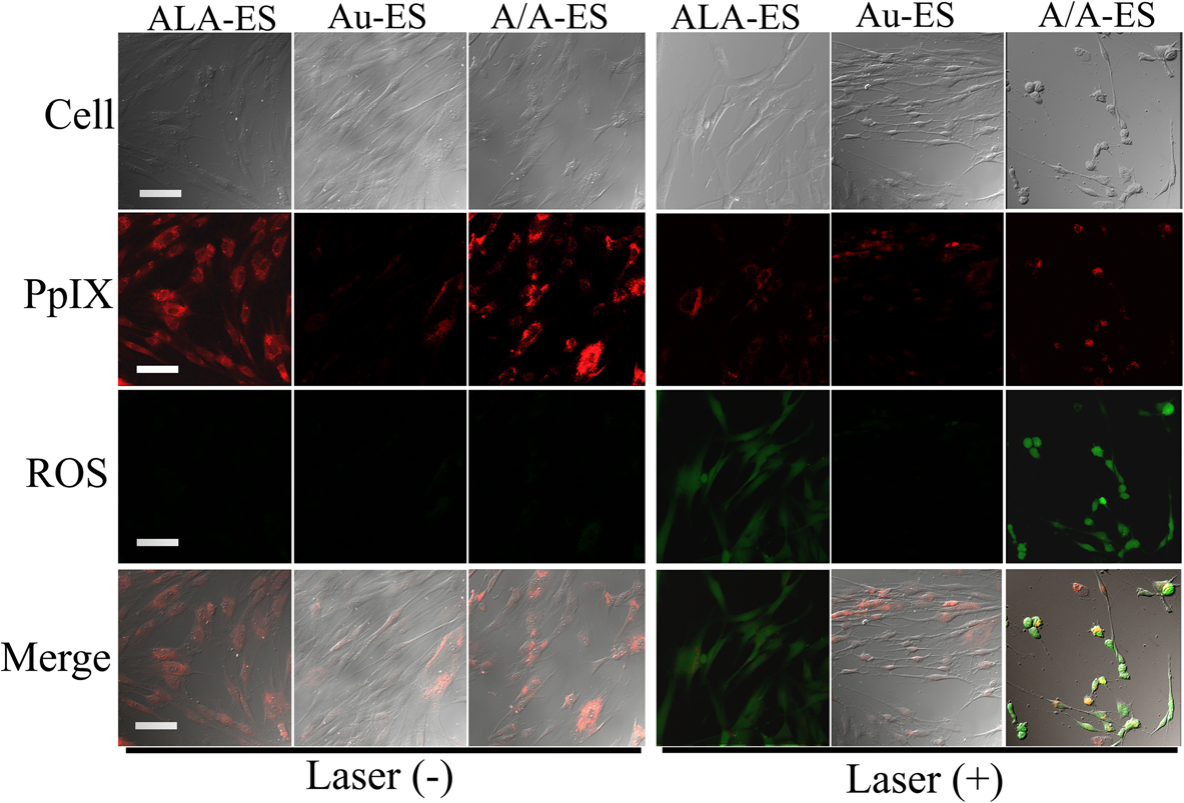
Confocal images of HSF treated with ALA-ES, Au-ES, and A/A-ES for 6 h and followed without/with irradiations. The scale bar is 100 μm

**Fig. 5:**
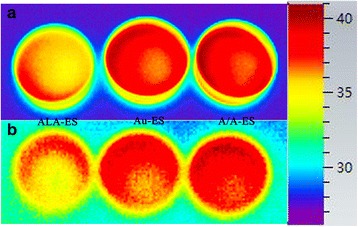
. Infrared microscopic imaging of HSF treated with ALA-ES, Au-ES, and A/A-ES for 6 h under (**a**) and after irradiation (**b**)

A/A-ES could overcome surface permeability barriers by the fusion of A/A-ES with HSF membrane and then liberate the ALA and AuNPs directly into the cell cytoplasm [32]. According to the mechanism of ALA-PDT, ALA released from A/A-ES could convert to PpIX in HSF cytoplasm. With laser, PpIX produced cytotoxic ROS leading to the cell apoptosis. Therefore, CLSM was used to study the accumulation of both PpIX and ROS in Fig. 4 [33, 34]. Before laser irradiation, the red fluorescence of PpIX was mainly distributed in the cytoplasm of HSF. PpIX in HSF treated by ALA-ES and A/A-ES were much more than the autologous PpIX in HSF treated by Au-ES. Moreover, ROS in all HSF was hardly found without laser irradiation, which was also reasonable. After laser irradiation, the PpIX intensities in HSF treated by ALA-ES and A/A-ES were reduced, and ROS in these cells could be easily found with strong intensity. Meanwhile, the HSF treated by Au-ES had no response in PpIX and ROS because they did not have enough autologous PpIX. Interestingly, A/A-ES could promote more ROS generation than ALA-ES in a comparison of ROS intensity, which was attributed to the AuNPs. Furthermore, the cell morphology also provided more information. The HSF treated by ALA-ES had the eumorphism, while the HSF treated by Au-ES displayed unhealthy protrusions from the plasma membrane. In contrast, the HSF treated by A/A-ES showed as protruding and retracting “blebs,” which was the feature of dying cells [35]. These differences in ROS generation and cell morphology were attributed to PTT based on AuNPs, which was also investigated by infrared imaging in Fig. 5. According to the mechanism of AuNPs-PTT, AuNPs in HSF cytoplasm could absorb 632 nm laser and generate enough heat to make the cells apoptosis or necrosis under irradiation. Therefore, the photothermal effects of ALA-ES, Au-ES, and A/A-ES were monitored by using an infrared thermal imaging camera. Compared ALA-ES, Au-ES, and A/A-ES had obviously higher temperature (41.3 °C for Au-ES and A/A-ES, 36.5 °C for ALA-ES) upon irradiation. After the laser was removed, temperatures of all quickly declined to a normal value in 1 min, suggesting that the laser irradiation-treatment could be safe [36]. Therefore, AuNPs loaded in ES could provide an effective PTT, which also provided by apoptosis and necrosis assay. To sum up, A/A-ES could enhance quantum yields of ROS and provide the photothermal effect to achieve an excellent efficiency of PDT/PTT synergistic treatment for HSF.

### Apoptosis and Necrosis Assay

**Fig. 6:**
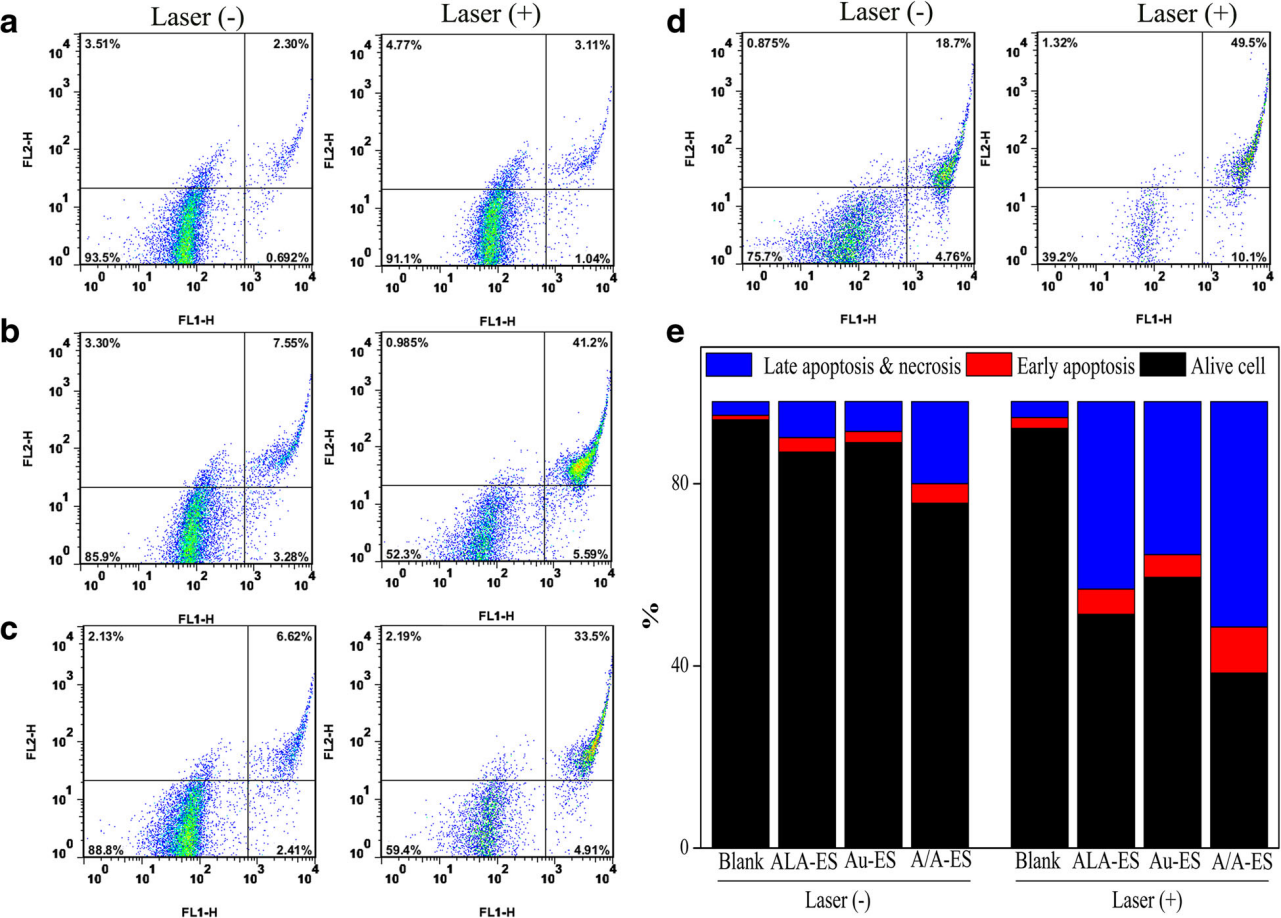
Apoptosis assay of HSF treated with ALA-ES (**b**), Au-ES (**c**), and A/A-ES without/with laser irradiation (**d**), and the statistical analysis of alive, early apoptotic and late apoptotic and necrosis (**e**). **a** was the control. Laser (-) and (+) mean without/with laser irradiation

The efficiency of PDT/PTT was further studied by the apoptosis and necrosis of HSF treated by ALA-ES, Au-ES, and A/A-ES under laser irradiation. An apoptosis assay was carried out by using the flow cytometry analysis of Annexin V-FITC and propidium iodide (PI) double staining (Fig. 6). A control results showed that laser irradiation did not affect cell viability (Fig. 6a). Before irradiating, ALA-ES, Au-ES, and A/A-ES displayed the good biocompatibility. After irradiation, the HSF treated by ALA-ES, Au-ES, and A/A-ES proportion of both necrosis and apoptosis had significant differences. Briefly, there was a highest fraction of both necrosis and apoptosis of HSF treated by A/A-ES, which was in consistence with the result of CLSM. In Fig. 6e, the statistical analysis of experiments revealed that necrotic cell death increased to 61.8% with the treatment with A/A-ES, indicating the A/A-ES had better synergistic PDT/PTT efficiency for HSF than the individual PDT (47.7% necrotic cell death) and PTT (24.3% necrotic cell death). Interestingly, the result also indicated that PDT played a more effective role in HS treatment compared with PTT, and AuNP-based PTT could help the PDT effect. These results might be explained as that A/A-ES could enhance quantum yields of ROS and provide the photothermal effect to achieve an excellent efficiency of PDT/PTT synergistic treatment for HSF. Although EE of ALA in A/A-ES was much lower than the one in ALA-ES (20 vs. 54%), there was similar necrotic cell death of both A/A-ES and ALA-ES (61.8 vs. 78%). This result could be explained from that A/A-ES could enhance quantum yields of ROS by photothermal effect and LSPR of AuNPs.

### Visualization of A/A-ES in HSF

**Fig. 7:**
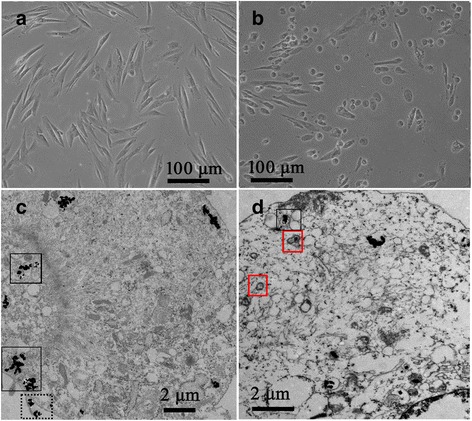
The light microscopy images of HSF treated with A/A-ES before (**a**) and after irradiation (**b**). TEM images of HSF treated with A/A-ES before (**c**) and after irradiation (**d**)

The detail changes of HSF morphology and structure caused by PDT/PTT also were investigated by both light microscopy and TEM in Fig. 7. Before irradiating, HSF treated with A/A-ES growth well, eumorphism firm adherence, indicating A/A-ES had excellent biocompatibility as expected (Fig. 7a). In their TEM image, besides various organelles in normal cytoplasm, treated HSF had a lot of AuNP aggregating in the cell cytoplasm (the blank frames in Fig. 7c). It could be explained that the fusion of A/A-ES with cell membranes could deliver more AuNPs and ALA into HSF. Therefore, AuNPs could act as the more effective photothermal source due to stronger plasmonic coupling effect and enhance the quantum yields of ROS by LSPR. Interestingly, shown in the dashed frame in Fig. 7c, some AuNPs were out of HSF due to exocytosis, which demonstrated the excellent biocompatibility of A/A-ES once more. After irradiation, HSF displayed the feature of dying cells, that is, the protrusions from the plasma membrane (Fig. 7b) [37]. Due to ROS and photothermal effect, the swelling mitochondria and the ruptured outer membrane, as the other indicators of HSF death, were found in HSF cytoplasm (Fig. 7d) [35]. Furthermore, ES was also found with its characteristic membrane structure (red frames in Fig. 7d). To sum up, A/A-ES could facilitate ALA and AuNPs penetrating into HSF and destroy HSF by synergistic PDT/PTT.

## Conclusions

Biocompatible A/A-ES was facilely prepared for in vitro synergistic PDT/PTT of HS by permeating into HS and destroying HSF. Utilizing ultrasonication, AuNPs were synthesized and loaded simultaneously in the absence of any toxic agents. A/A-ES had strong absorbance in 600–650 nm as the plasmonic coupling effect between neighboring AuNPs in the ES with a high EE for ALA (ca. 20%). In vitro transdermal penetrability study demonstrated that A/A-ES was a highly efficient drug carrier to enhance both ALA and AuNP penetration into HS tissue*.* In vitro PDT/PTT for HSF indicated that A/A-ES could enhance quantum yields of ROS by photothermal effect and LSPR of AuNPs, causing a high level of cell apoptosis or necrosis. In a word, biocompatible A/A-ES had a better synergistic PDT/PTT efficiency for HSF than the individual PDT and PTT, encouraging perspective for treatment of HS. Further work will focus on the in vivo study of synergistic PDT/PTT for HS in scar models, and the relevant work is ongoing.

## Experiments and Methods

### The Preparation of A/A-ES

**Scheme 1:**
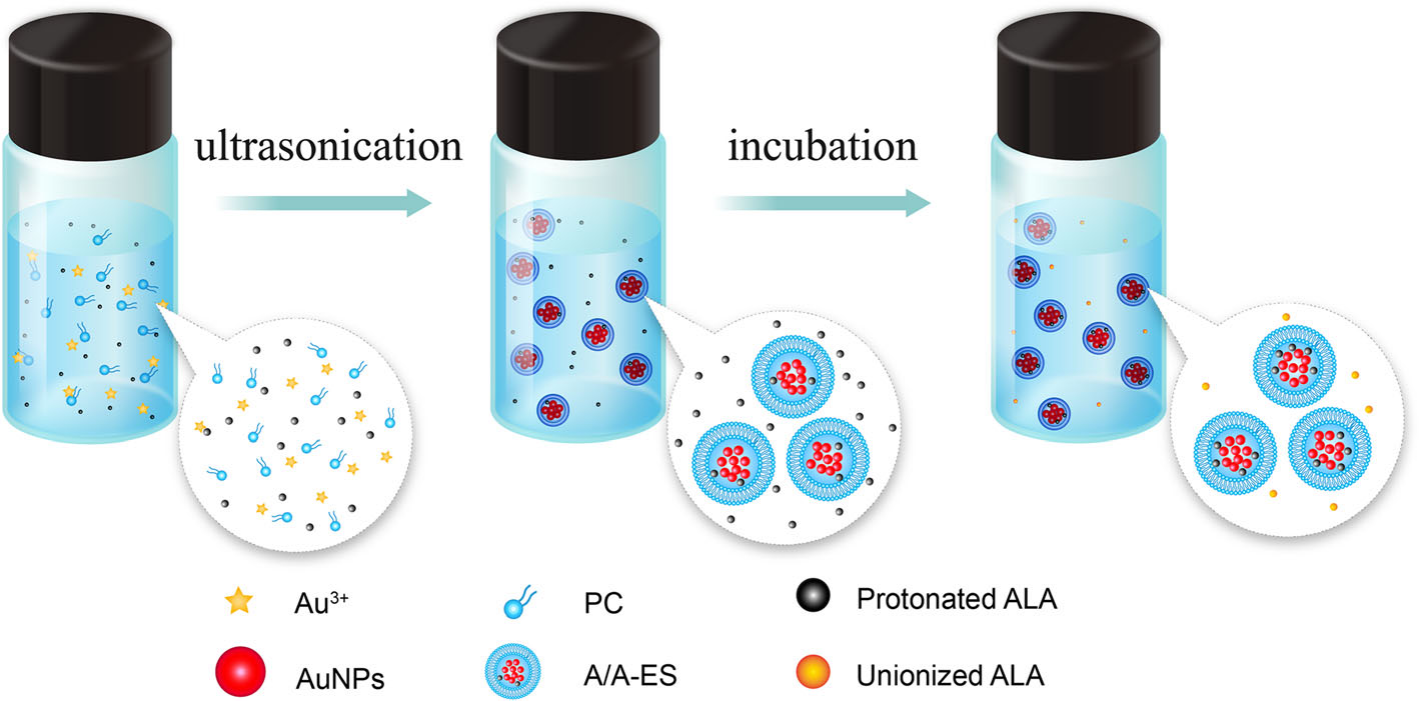
Schematic diagram of preparing A/A-ES

One hundred eighty milligrams of phosphatidylcholine (PC, 95.8% soybean lecithin, Lipoid GmbH, Germany) dissolved in 1.8 mL CH_3_CH_2_OH, 0.6 mL HAuCl_4_ (10 mM, Aladdin, Shanghai, China), and 3.6 mL ALA-citrate buffer solution (CBS, 0.01 M, 12 mg ALA, pH 4.0), in turn, were added into PC solution by dropwise. The mixture was stirred at 700 rpm for 10 min to prepare precursor solution. As shown in Scheme 1, the precursor solution was put in an ultrasonic environment at 200 W for 30 min, until it was brilliant wine red color. Then, the reaction solution was carried out with a centrifuge (8000 rpm, 20 min) to remove the residual HAuCl_4_ and PC. Last, the deposition was re-dispersed in 3 ml ALA hydroalcoholic solution (ALA-HA, 2 mg/ml ALA, 30% ethanol) and incubated by a transmembrane pH gradient active loading method according to our prior work [13]. In incubation, a plenty of exterior unionized ALA diffused through the ES bilayers into the internal acidic aqueous core of ES, and then, they were protonated and entrapped in ES. After incubation, A/A-ES has been prepared. In this work, ALA-ES was prepared according to our prior work with the same ALA concentration as A/A-ES. AuNP-loaded ES (Au-ES) was prepared as A/A-ES without ALA with the same AuNP concentration as A/A-ES.

### The Characterization of A/A-ES

A/A-ES were negatively stained with phosphotungstic acid (1.5 wt%) and then observed by transmission electron microscope (TEM, JEOL, Japan, accelerating voltage of 120 kV). A/A-ES was also examined by a scanning electron microscopy (SEM, JEOL, Japan, accelerating voltage of 10 kV). The A/A-ES size distribution was determined by dynamic light scattering (DLS) analysis in a NiComp 380ZLS inspection system (Nicomp, USA). ALA was determined by a fluoresceamine derivatization approach, and the detail was shown in Additional file 1. The entrapment efficiency (EE) of ALA determined by an ultrafiltration method was shown in Additional file 1. Finally, UV-Vis spectra were carried out on a Varian Cary 50 UV-Vis spectrophotometer (Perkin Elmer, USA).

### In Vitro Penetrability Study by Franz Diffusion Cells

The penetrability study of A/A-ES was carried out by using Franz diffusion cells with 2.8 cm^2^ effective permeation area. The receptor cells including donor and receptor compartments were maintained at 37 °C by circulating water bath. HS tissues were collected with informed consents at Shanghai Ninth People’s Hospital and the ethical guidelines of the 1975 Declaration of Helsinki approved by Shanghai Ninth People’s Hospital. Fresh HS tissue without fatty tissue (less than 24 h after excision) was mounted on a receptor compartment with stratum corneum upward to the donor compartment. One milliliter of A/A-ES was added into donor compartment, and then, donor compartment was covered by parafilm to prevent evaporation. After penetration with different time, HS tissues were washed promptly to remove residual A/A-ES on HS surface. To accumulate the retention amount of ALA and AuNPs in HS, HS tissues were cut to small pieces, and ALA in HS tissues was extracted by dialysis in PBS for 24 h. The extract solutions were analyzed for retention amount of ALA in HS tissue. The HS tissues retained in dialysis bags were also analyzed for retention amount of AuNP by inductively coupled plasma-mass spectrometry (ICP-MS). After permeated by ALA-ES for 2 h, HS tissue was washed, prefixed, dehydrated, infiltrated, and post fixed. After embedded in epoxy resins, they were cut as ultrasections (50 nm thickness, perpendicular to epidermis) and observed by using TEM at an accelerating voltage of 120 kV.

### In Vitro PDT/PTT for HSF

#### Cell Culture

HSF was isolated and cultured by a common method as follows: The fresh HS tissue pieces (1 mm^3^, less than 6 h after excision) were digested by using collagenase type I (Invitrogen, USA) to achieve single cell suspension. The HSF grew in Dulbecco’s Modified Eagle Medium (DMEM, Invitrogen, USA) containing 10% fetal bovine serum (FBS, Gibco, USA) at 37 °C and 5% CO_2_. Culture medium should be changed every 3 days, and cells were passaged when 80% confluent. The passaging two and three cells were used in the following experiments.

### Biocompatibility Assay

In the evaluation of the biocompatibility of A/A-ES, HSF were seeded in 96-well plates at 2 × 10^3^ cells/well. The culture medium was replaced with FBS-free medium and freshly prepared ALA-ES, Au-ES, and A/A-ES in different concentrations, respectively. After 12 h, the cell viability was measured using a cell counting kit-8 (CCK-8, Dojindo, Japan) following the manufacturer’s instructions.

### PDT/PTT Procedure

HSF were seeded in 12-well plates at 4 × 10^4^ cells/well. After 12 h, culture mediums, respectively, containing fresh prepared ALA-ES, Au-ES, and A/A-ES (14%, *v*/*v*), were replaced with the FBS-free medium for 6 h. After treatment, HSF was washed with PBS and incubated in culture medium for 1 h. Then, they were irradiated by He-Ne laser (632 nm wavelength, 40 mW/cm^2^, Shanghai Institute of Laser Technology, China) with 20 min. Then, culture medium was replaced with fresh DMEM containing 10% FBS for another 24 h in preparation for subsequent experiments. Furthermore, HSF treated with A/A-ES and irradiation was prefixed, dehydrated, and embedded to prepare ultrasections for TEM examination.

### Intracellular PpIX and ROS Generation Assay

Intracellular PpIX accumulation and ROS generation in HSF were detected by using confocal laser scanning microscopy (CLSM, Leica TCS SP5, Germany). The ROS generation assay was performed using a DCFH-DA and followed the manufacturer’s instructions. The coverslip with cells was mounted on a glass slide and observed at 405 nm excitation/635 nm emission for PpIX and 488 nm excitation/560 nm emission for ROS. All data was analyzed by LAS AF software.

### Apoptosis and Necrosis Assay

The apoptosis and necrosis of HSF were analyzed by flow cytometry after double staining Annexin V-FITC and propidium iodide (PI) double staining. The samples were prepared according to the protocol of Annexin V-FITC/PI apoptosis detection kit and then analyzed by BD FACSCalibur (BD Biosciences, Mountain View, USA). The data analysis was performed with FlowJo 7.6 software.

### Statistical Analysis

Data were presented as mean ± SD unless otherwise stated. Statistical significance was determined using a two-tailed student’s test (*P* < 0.05) unless otherwise stated.

## Additional file

Additional file 1: Figure S1. The TEM images of A/A-ES containing different AuNPs. (DOCX 1028 kb)

## Abbreviations

A/A-ES: 5-Aminolevulinic acid/Au nanoparticle-loaded ethosomal vesicle
ALA: 5-Aminolevulinic acid
ALA-ES: ALA-loaded ES
ALA-PDT: ALA-based PDT
Au-ES: AuNP-loaded ES
AuNPs: Au nanoparticles
CLSM: Confocal laser scanning microscopy
DLS: Dynamic light scattering
DMEM: Dulbecco’s Modified Eagle Medium
EE: Entrapment efficiency
ES: Ethosomal vesicles
FBS: Fetal bovine serum
HS: Hypertrophic scar
HSF: Hypertrophic scar fibroblasts
ICP-MS: Inductively coupled plasma-mass spectrometry
LSPR: Localized surface plasmon resonance
PDT: Photodynamic therapy
PDT/PTT: Photodynamic/photothermal therapy
PpIX: Protoporphyrin IX
PTT: Photothermal therapy
ROS: Reactive oxygen species
SEM: Scanning electron microscopy
TEM: Transmission electron microscope

## References

[1] Sogabe Y, Akimoto S, Abe M, Ishikawa O, Takagi Y, Imokawa G (2002). Functions of the stratum corneum in systemic sclerosis as distinct from hypertrophic scar and keloid functions. J Dermatol Sci.

[2] Alster TS, Handrick C (2000). Laser treatment of hypertrophic scars, keloids, and striae. Semin Cutan Med Surg.

[3] Ehrlich HP, Desmouliere A, Diegelmann RF, Cohen IK, Compton CC, Garner WL (1994). Morphological and immunochemical differences between keloid and hypertrophic scar. Am J Pathol.

[4] Vrijman C, van Drooge AM, Limpens J, Bos JD, van der Veen JPW, Spuls PI (2011). Laser and intense pulsed light therapy for the treatment of hypertrophic scars: a systematic review. Brit J Dermatol..

[5] Li-Tsang CW, Feng B, Huang L, Liu X, Shu B, Chan YT (2015). A histological study on the effect of pressure therapy on the activities of myofibroblasts and keratinocytes in hypertrophic scar tissues after burn. Burns.

[6] Vrijman C, van Drooge AM, Limpens J, Bos JD, van der Veen JP, Spuls PI (2011). Laser and intense pulsed light therapy for the treatment of hypertrophic scars: a systematic review. Bri J dermatol.

[7] Juckett G, Hartman-Adams H (2009). Management of keloids and hypertrophic scars. Am Fam Physician.

[8] Chang M, Ma X, Ouyang T, Lin J, Liu J, Xiao Y (2015). Potential molecular mechanisms involved in 5-Aminolevulinic acid–based photodynamic therapy against human hypertrophic scars. Plast Reconstr Surg.

[9] Karrer S, Bosserhoff A, Weiderer P, Landthaler M, Szeimies RM (2004). Keratinocyte-derived cytokines after photodynamic therapy and their paracrine induction of matrix metalloproteinases in fibroblasts. Bri J Dermatol.

[10] Wang Q, Dong Y, Geng S, Su H, Ge W, Zhen C (2014). Photodynamic therapy inhibits the formation of hypertrophic scars in rabbit ears by regulating metalloproteinases and tissue inhibitor of metalloproteinase-1. Clin Expe Dermatol.

[11] Morilla MJ, Romero EL (2016). Carrier deformability in drug delivery. Curr Pharm Des.

[12] Dayan N, Touitou E (2000). Carriers for skin delivery of trihexyphenidyl HCl: ethosomes vs. liposomes. Biomaterials.

[13] Zhang Z, Chen Y, Xu H, Wo Y, Zhang Z, Liu Y (2016). 5-Aminolevulinic acid loaded ethosomal vesicles with high entrapment efficiency for in vitro topical transdermal delivery and photodynamic therapy of hypertrophic scars. Nano.

[14] Chen S, Bao C, Zhang C, Yang Y, Wang K (2016). EGFR antibody conjugated bimetallic au@Ag nanorods for enhanced SERS-based tumor boundary identification, targeted photoacoustic imaging and photothermal therapy. Nano Biomed. Eng..

[15] Ravalika V, Sailaja A (2017). Formulation and evaluation of etoricoxib niosomes by thin film hydration technique and ether injection method. Nano Biomed. Eng..

[16] Lal S, Clare SE, Halas NJ (2008). Nanoshell-enabled photothermal cancer therapy: impending clinical impact. Accounts Chem Res.

[17] Mamalis A, Koo E, Sckisel GD, Siegel DM, Jagdeo J (2016). Temperature-dependent impact of thermal aminolaevulinic acid photodynamic therapy on apoptosis and reactive oxygen species generation in human dermal fibroblasts. Brit J Dermatol.

[18] Li S, Luan W, Tu S, Shan Y (2011). Au-Ag gradient alloy nanoparticles with extended surface plasmon resonance wavelength: synthesis via microreaction. Nano Biomed Eng.

[19] Zhang ZX, Wang SJ, Xu H, Wang B, Yao CP (2015) Role of 5-aminolevulinic acid-conjugated gold nanoparticles for photodynamic therapy of cancer. J Biomed Opt 20(5) 10.1117/1.JBO.20.5.051043 26021715

[20] Lin J, Wang S, Huang P, Wang Z, Chen S, Niu G (2013). Photosensitizer-loaded gold vesicles with strong plasmonic coupling effect for imaging-guided photothermal/photodynamic therapy. ACS Nano.

[21] Yan X, Hu H, Lin J, Jin AJ, Niu G, Zhang S (2015). Optical and photoacoustic dual-modality imaging guided synergistic photodynamic/photothermal therapies. Nano.

[22] Rao YF, Zheng FY, Zhang XG, Gao JQ, Liang WQ (2008). In vitro percutaneous permeation and skin accumulation of finasteride using vesicular ethosomal carriers. AAPS PharmSciTech.

[23] Chiu LL, Sun CH, Yeh AT, Torkian B, Karamzadeh A, Tromberg B (2005). Photodynamic therapy on keloid fibroblasts in tissue-engineered keratinocyte-fibroblast co-culture. Laser Surg Med.

[24] Torchia D (2009). Photodynamic therapy on intact and/or thick skin. Clin Expe Dermatol.

[25] Pegoraro C, MacNeil S, Battaglia G (2012). Transdermal drug delivery: from micro to nano. Nano.

[26] HX X, Zeiger BW, Suslick KS (2013). Sonochemical synthesis of nanomaterials. Chem Soc Rev.

[27] Kosobe T, Moriyama E, Tokuoka Y, Kawashima N (2005). Size and surface charge effect of 5-aminolevulinic acid-containing liposomes on photodynamic therapy for cultivated cancer cells. Drug Dev Ind Pharm.

[28] Touitou E, Dayan N, Bergelson L, Godin B, Eliaz M (2000). Ethosomes––novel vesicular carriers for enhanced delivery: characterization and skin penetration properties. J Control Release.

[29] Touitou E, Ainbinde D (2014). 7. Ethosomes––an innovative carrier for enhanced delivery into and across the skin: original research article: ethosomes––novel vesicular carriers for enhanced delivery: characterization skin penetration properties, 2000. J Control Release.

[30] Liu Y, Yang M, Zhang J, Zhi X, Li C, Zhang C (2016). Human induced pluripotent stem cells for tumor targeted delivery of gold nanorods and enhanced photothermal therapy. ACS Nano.

[31] Hou W, Xia F, Alfranca G, Yan H, Zhi X, Liu Y (2017). Nanoparticles for multi-modality cancer diagnosis: simple protocol for self-assembly of gold nanoclusters mediated by gadolinium ions. Biomaterials.

[32] Godin B, Touitou E (2004). Mechanism of bacitracin permeation enhancement through the skin and cellular membranes from an ethosomal carrier. J Control Release.

[33] Couleaud P, Morosini V, Frochot C, Richeter S, Raehm L, Durand JO (2010). Silica-based nanoparticles for photodynamic therapy applications. Nano.

[34] Wang LY, Shi XY, Yang CS, Huang DM (2013). Versatile RBC-derived vesicles as nanoparticle vector of photosensitizers for photodynamic therapy. Nano.

[35] Huang P, Li Z, Lin J, Yang D, Gao G, Xu C (2011). Photosensitizer-conjugated magnetic nanoparticles for in vivo simultaneous magnetofluorescent imaging and targeting therapy. Biomaterials.

[36] Song CW, Park HJ, Lee CK, Griffin R (2005). Implications of increased tumor blood flow and oxygenation caused by mild temperature hyperthermia in tumor treatment. Int J Hyper.

[37] Hacker G (2000). The morphology of apoptosis. Cell Tissue Res.

