# Supplementary material for: Biocompatible 5-Aminolevulinic Acid/Au Nanoparticle-Loaded Ethosomal Vesicles for In Vitro Transdermal Synergistic Photodynamic/Photothermal Therapy of Hypertrophic Scars
Source: Nanoscale Res Lett. 2017 Dec 15;12:622. doi: 10.1186/s11671-017-2389-x (PMC5732126; doi:10.1186/s11671-017-2389-x)
Supplement: Additional file 1: Figure S1. — The TEM images of A/A-ES containing different AuNPs. (DOCX 1028 kb) [file 11671_2017_2389_MOESM1_ESM.docx]

**[Biocompatible 5-Aminolevulinic](http://www.sciencedirect.com/science/article/pii/S0021967312004694)****[Acid/Au Nanoparticles Loaded Ethosomal Vesicles for](http://www.sciencedirect.com/science/article/pii/S0021967312004694)** ***in Vitro*** **Transdermal Synergistic Photodynamic/Photothermal Therapy of Hypertrophic Scars**

Zheng Zhang^1※,*^, Yunsheng Chen^1,2※^, Jiayue Ding^3※^, Chunlei Zhang^2^, Amin Zhang^2^, Dangnong He^4^, Yixin Zhang^1,4, *^

1 Department of Plastic and Reconstructive Surgery, Shanghai Ninth People's Hospital, School of Medicine, Shanghai Jiao Tong University, 639 Zhizaoju Rd, Shanghai 200011, P.R. China;

2 Institute of Nano Biomedicine and Engineering, Shanghai Engineering Research Center for Intelligent Instrument for Diagnosis and Therapy, School of Biomedicine Engineering, 800 Dongchuan Rd, Shanghai Jiao Tong University, Shanghai 200240, P.R. China;

3 Department of Plastic Surgery, Lishui People Hospital, 15 dazong Rd, Lishui, Zhejiang, 323000, China

4 Shanghai National Engineering Research Center for Nanotechnology, 245 Jiachuan Road, Shanghai 200237, PR China

※These authors contributed equally to this work.

* Authors to whom correspondence should be addressed; E-Mail: [Zhangzheng958@163.com](mailto:Zhangzheng958@163.com), Zhangyixin6688@163.com.

**S1 Figure captions**

**Figure S1** The TEM images of A/A-ES containing different AuNPs.

**S2 Experimental details**

**S2.1 Quantitative analytical of ALA using 96-well microplate**

ALA was rapidly and accurately detected by a modified fluoresceamine derivatization approach using 96-well micro-plates. Briefly, 12 μL sample, 270 μL fluorescamine dissolved in acetonitrile (0.1%, w/v), 45 μL borate buffer solution, and 570 μL water were added into a microfuge tube in sequence. The mixture was vortexed for 5 s and allowed to react in dark environment for 5 min. 300 μL reacted mixtures were transferred to the wells of a 96-well flat-bottomed microplate (Corning, USA) and their fluorescence intensities were obtained by a Synergy H4 hybrid reader (Bio-Tek, USA) with 380 nm excitation/ 480 nm emission. The fluorescence intensity (RFI) at 480 nm presented a good linearity with ALA concentration.

**S2.1 Determination of entrapment efficiency (EE) of ALA**

The EE of ALA was also determined by an ultrafiltration method. 20 μL A/A-ES suspension was diluted to 200 μL with PBS, placed in an Ultra-0.5 filters (molecular weight cut-off 30 KDa, Millipore, USA) and centrifuged at the speed of 5000 rpm for 30 min to separate the untrapped ALA from A/A-ES. Then the ultrafiltrates were carefully collected and diluted to 1 mL with PBS, and the ALA amount in ultrafiltrate (Q_f_) were determined. Furthermore, the 20 μL A/A-ES suspension was added into 180μL ethanol to destroy all ES, and the total ALA amount added (Q_t_) was determined. Then EE equation was adjusted as Eq. 1:

EE = (1 − Q_f_ / Q_t_) × 100%. (1)


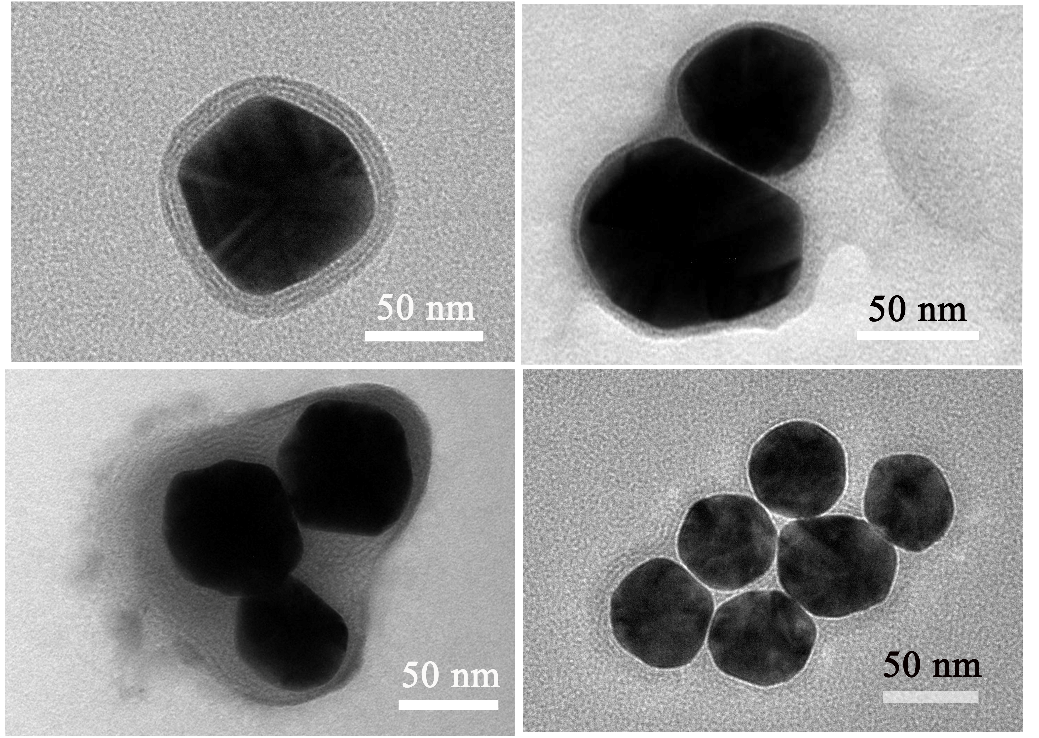


**Figure S1**
